# Efficient clinical data analysis for prediction of coal workers' pneumoconiosis using machine learning algorithms

**DOI:** 10.1111/crj.13657

**Published:** 2023-06-28

**Authors:** Hantian Dong, Biaokai Zhu, Xiaomei Kong, Xinri Zhang

**Affiliations:** ^1^ Department of Geriatric Diseases First Hospital of Shanxi Medical University Taiyuan Shanxi People's Republic of China; ^2^ National Health Commission Key Laboratory of Pneumoconiosis, Shanxi Province Key Laboratory of Respiratory Diseases Department of Pulmonary and Critical Care Medicine, First Hospital of Shanxi Medical University Taiyuan Shanxi People's Republic of China; ^3^ Network Security Department Shanxi Police College Taiyuan Shanxi People's Republic of China

**Keywords:** coal workers' pneumoconiosis clinical prediction, feature selection, machine learning

## Abstract

**Purpose:**

The purpose of this study is to propose an efficient coal workers' pneumoconiosis (CWP) clinical prediction system and put it into clinical use for clinical diagnosis of pneumoconiosis.

**Methods:**

Patients with CWP and dust‐exposed workers who were enrolled from August 2021 to December 2021 were included in this study. Firstly, we chose the embedded method through using three feature selection approaches to perform the prediction analysis. Then, we performed the machine learning algorithms as the model backbone and combined them with three feature selection methods, respectively, to determine the optimal predictive model for CWP.

**Results:**

Through applying three feature selection approaches based on machine learning algorithms, it was found that AaDO_2_ and some pulmonary function indicators played an important role in prediction for identifying CWP of early stage. The support vector machine (SVM) algorithm was proved as the optimal machine learning model for predicting CWP, with the ROC curves obtained from three feature selection methods using SVM algorithm whose AUC values of 97.78%, 93.7%, and 95.56%, respectively.

**Conclusion:**

We developed the optimal model (SVM algorithm) through comparisons and analyses among the performances of different models for the prediction of CWP as a clinical application.

## Graphical abstract

After close data analysis and careful evaluation of machine learning algorithms, clinical indicators belonged to significant predictors of pulmonary disease diagnosis. We chose the embedded method using three feature selection approaches to perform the prediction analysis, then performed machine learning algorithms as the model backbones, and combined them with three feature selection methods, respectively, to determine the optimal predictive model.

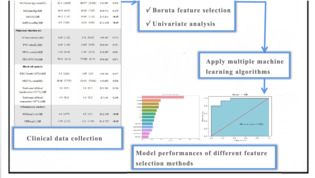

## 1 INTRODUCTION

Coal workers' pneumoconiosis (CWP) is a damaging kind of chronic occupational lung disease that results from inhalation of mineral dust, remaining one of the most common occupational diseases in China, accounting for about 50% of the total number of newly confirmed cases of diagnosed pneumoconiosis reported every year.^1^, ^2^, ^3^, ^4^, ^5^ No specific therapy to effectively delay the disease progression of CWP has been developed. Hence, improving the early diagnosis rates of CWP is a crucial issue.

The etiology and pathogenesis of CWP remain to be systematically elucidated, while no clinically available early diagnosis can distinguish CWP with early stage from dust‐exposed workers up to now.^6^ According to International Labour Organization (ILO) guidelines, chest X‐rays are essential for the early screening, staging, and diagnosing of pneumoconiosis. However, relying solely on diagnostic imaging methods may result in inaccurate clinical diagnoses, and combining serological tests may provide additional evidence to characterize CWP patients comprehensively.^7^, ^8^

CWP contributes to the development or aggravation of pulmonary infections and inflammatory diseases of unknown etiology that can result from comprehensive factors working together, such as pneumonia, interstitial lung diseases, emphysema, liver injury, kidney injury, kidney injury, and tumors in multiple organs.^9^, ^10^, ^11^, ^12^, ^13^, ^14^ The above inflammatory lung diseases might lead to activation of the blood coagulation system,^15^ and coagulation‐inflammation interactions might occur in CWP. As such, we hypothesized that coagulation function and inflammatory markers might help predict the risk of CWP. Blood cell analysis and serum tumor markers, as highly sensitive and specific diagnostic indicators for above inflammatory lung diseases and early‐stage tumors, might also reflect the inflammatory status of early‐stage CWP. It thus makes sense to evaluate its prediction clinically.

However, no currently available clinical indicator or system can provide sufficiently accurate predictions for disease progression in CWP patients of the early phase.^16^ Therefore, developing and validating sensitive and specific clinical indicators to effectively predict the progression of CWP in the early phase is essential. The objective of this research was the development of a computational tool for predicting the risk of CWP with early stage in dust–exposed workers from large amounts of clinical indicators, which have shown that there were differences between patients confirmed CWP and dust–exposed workers, including arterial blood gas analysis, pulmonary function test, blood cell analysis, inflammatory markers, blood biochemical parameters, coagulation function, and serum tumor markers.

Advances in artificial intelligence (AI) and mainly in machine learning (ML) have been rapidly gaining importance in assisting a clinical practice in diagnostic decision‐making.^17^, ^18^, ^19^, ^20^, ^21^ With respect to pneumoconiosis application, AI algorithms have shown remarkable success in medical image analysis, especially in detecting imaging features of pneumoconiosis.^22^, ^23^ However, an ML algorithm for the prediction of pneumoconiosis clinically is lacking.

In this study, we aimed to predict CWP from the secondary prevention perspective, suggesting a novel way of understanding the diagnostic classification of pneumoconiosis in a clinical environment at an early phase by assessing the conventional clinical indicators. We proposed an efficient and accurate CWP clinical prediction system after the comparative analysis of the performances of different machine learning algorithms. Thus, ideal indicators with better sensitivity and specificity could be identified and then put into clinical use for the clinical diagnosis of pneumoconiosis.

## 2 MATERIALS AND METHODS

### 2.1 Patients source and clinical data collection

**FIGURE 1:**
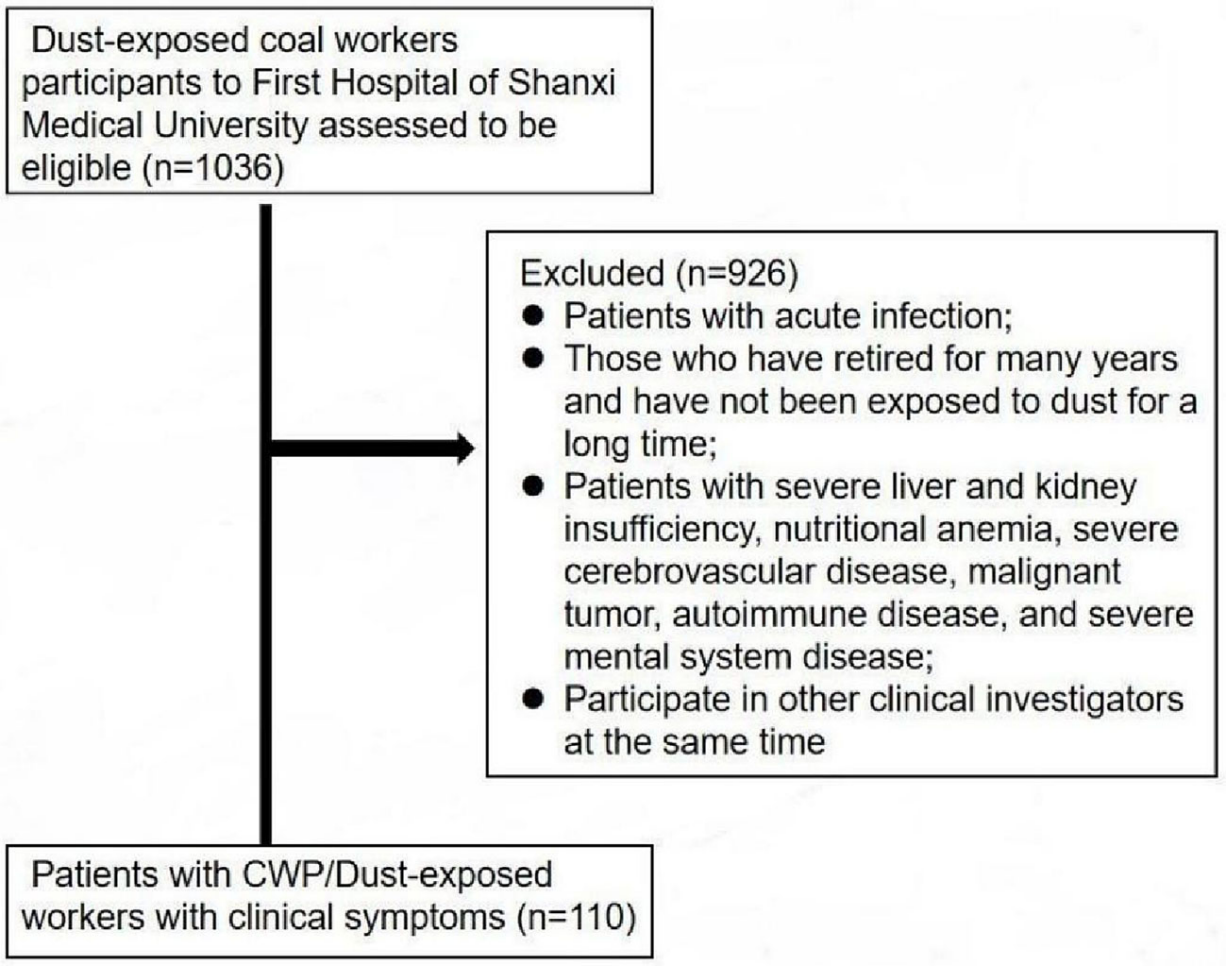
Flowchart indicating inclusion criteria for identification of patients with CWP and dust‐exposed workers with clinical symptoms.

During 28 August 2021 until 12 December 2021, 52 patients with CWP and 58 dust‐exposed workers, belonging to male patients aged 33–70 years with cough, dyspnoea, or other symptoms, were enrolled in this study (shown in Figure 1). Since CWP, an occupational disease, is relatively uncommon and an exploratory study designed, we did not calculate a standard sample size. The clinical data with significant differences, including arterial blood gas analysis, pulmonary function test, blood cell analysis, inflammatory markers, blood biochemical parameters, coagulation function, and serum tumor markers, are presented in Table 1 (for full list of clinical data, see Table S1). This dataset consisted of 62 collected clinical parameters, all belonging to continuous variables. Prior to statistical analyses, the data were reviewed for outliers and missing data, and no outliers were identified.

**TABLE 1:** Clinical data of patients in the group with CWP and with dust‐exposed workers.

	Dust‐exposed workers/control group (*n* = 58)	CWP Stage I (*n* = 52)	Statistic	*p*‐Value
Arterial blood gas analysis				
pH, mean (SD)	7.378 (0.021)	7.381 (0.019)	t = 0.772	0.442
PaCO_2_ (mmHg), mean (SD)	41.2 (2.694)	40.977 (2.918)	t = 0.005	0.996
PaO_2_ (mmHg), IQR	89.9 (9.07)	85.95 (7.47)	Z = 0.714	0.475
SaO_2_ (%), IQR	96.3 (1.15)	95.85 (1.32)	Z = 3.414	<0.05
AaDO_2_ (mmHg), IQR	8.9 (7.09)	13.51 (6.43)	Z = 2.128	<0.05
Pulmonary function test				
VCmax‐value (L), IQR	4.19 (1.02)	3.72 (0.63)	Z = 0.383	0.702
FVC‐value (L), IQR	4.09 (1.06)	3.635 (0.55)	Z = 0.344	0.731
FEV1‐value (L), IQR	3.14 (0.94)	2.765 (0.53)	Z = 0.467	0.641
FEV1/FVC (%), IQR	79.34 (6.41)	77.595 (8.17)	Z = 0.163	0.871
Blood cell analysis				
WBC count (×10^9^/L), IQR	5.5 (2.02)	6.05 (2.0)	Z = 0.744	0.457
NEUT%, mean (SD)	58.98 (7.775)	63.431 (7.814)	t = 0.003	0.998
Total count of blood lymphocytes (×10^12^/L), IQR	1.6 (0.5)	1.6 (0.7)	Z = 1.243	0.214
Total count of blood monocytes (×10^12^/L), IQR	0.4 (0.2)	0.4 (0.2)	Z = 1.14	0.254
Inflammatory markers				
ESR (mm/1_st_ h), IQR	4.0 (4.75)	6.5 (4.0)	Z = 2.349	<0.05
CRP (mg/L), IQR	2.59 (2.02)	3.12 (1.69)	Z = 1.725	<0.05
Blood biochemical parameters				
ALT (IU/L), IQR	29.0 (14.75)	21 (10.25)	Z = 2.635	<0.05
AST (IU/L), IQR	27.0 (8.5)	22 (5.25)	Z = 3.242	<0.05
GGT (IU/L), IQR	31 (20.25)	38.5 (73.25)	Z = 2.951	<0.05
CK (IU/L), IQR	110.0 (61.75)	96.5 (30.5)	Z = 1.848	<0.05
Ca^2+^ (mmol/L), IQR	1.125 (0.03)	1.1 (0.12)	Z = 2.335	<0.05
BNP (pg/mL), IQR	9.0 (10.5)	12 (12.0)	Z = 1.959	<0.05
HbA1c (%), IQR	4.63 (0.46)	5.44 (0.7)	Z = 2.367	<0.05
Coagulation function				
PT (s), IQR	11.6 (0.7)	11.9 (0.5)	Z = 0.795	0.426
PTA (%), IQR	93.8 (13.6)	88.5 (8.6)	Z = 0.7	0.484
INR, IQR	1.01 (0.07)	1.04 (0.04)	Z = 2.777	<0.05
D‐dimer (mg/L), IQR	0.18 (0.19)	0.26 (0.25)	Z = 2.623	<0.05
Serum tumor markers				
CEA (ng/mL), IQR	2.12 (2.31)	2.125 (2.05)	Z = 1.508	0.132
SCC (ug/L), IQR	0.71 (0.26)	0.73 (0.42)	Z = 1.359	0.174
CA19‐9 (U/mL), IQR	5.27 (3.79)	9.195 (9.65)	Z = 3.258	<0.05
CA125 (U/mL), IQR	11.33 (3.23)	10.925 (3.33)	Z = 1.227	0.220

*Note*: Bold characters represent statistical significance. Values are given as median (lower quartile, upper quartile) or *n* (percent).

Abbreviations: AaDO_2_, alveolar‐arterial oxygen difference; ALT, alanine transaminase; AST, aspartate aminotransferase; BNP, B‐natriuretic peptide; CA125, carbohydrate antigen 125; CA19‐9, carbohydrate antigen 19‐9; CEA, carcinoembryonic antigen; CK, creatine kinase; CRP, C‐reactive protein; CWP, coal workers' pneumoconiosis; CYFRA21‐1, cytokeratin 19 fragment antigen 21‐1; ESR, Erythrocyte sedimentation rate; FEV1‐value, value of the forced expiratory volume in the first second; FVC‐value, value of forced vital capacity; GGT, gamma glutamyl transpeptidase; HbA1c, hemoglobin A1c; INR, international normalized ratio; IQR, interquartile range; NEUT%, percentage of neutrophils; NSE, neuron‐specific enolase; PaCO_2_, arterial carbon dioxide tension; PaO_2_, arterial partial pressure of oxygen; PH, hydrogen ion concentration; PT, prothrombin time; PTA, prothrombin time activity; SaO_2_, arterial blood oxygen saturation; SCC, squamous cell carcinoma antigen; VCmax‐value, max value of vital capacity; WBC, white blood cell.

All the patients involved in the study provided written informed consent forms. The Research Ethics Committees of the First Hospital of Shanxi Medical University provided ethical approval for the study (reference no. 2020 K‐K104). In addition, this study was conducted as a diagnostic test and registered in the China Clinical Trial Registration Center (ChiCTR2100050379). The diagnostic criteria of patients with CWP (Stage I) were determined mainly from the typical imaging features of chest X‐ray (according to GBZ70‐2015), along with exposure duration history.

### 2.2 Feature selection

Owing to the amount of data and the number of features in this study, these variables were high‐dimensional, which posed an overfitting challenge for data analysis of machine learning models—accordingly, the smaller the feature variables, the more energetically favorable the analyses. As a data reduction strategy, feature selection aims to build more straightforward and comprehensible models, maximize data reliability, and conduct understandable and clean data.

Among these feature variables, applying an effective method to remove irrelevant or redundant features is crucial, especially since there is a paucity of clinical research on CWP. Current approaches for feature selection can be roughly categorized into three major classes: filter, wrapper, and embedded. In our research, we choose the embedded method by using three feature selection approaches (Lasso CV regression, Boruta feature selection, and univariate analysis) to perform the prediction analysis.

### 2.3 Machine learning model

Machine learning algorithms usually learn features from data through probability theory and can be classified into two main categories: supervised learning (labeled dataset) and unsupervised learning (unlabeled dataset). Unlike unsupervised ML algorithms, supervised learning can evaluate the prediction results from labeled cases. Given the limited training data available and our study aims for building classifiers for diagnostic classification, we performed the supervised learning algorithms as the model backbone and combined them with three feature selection methods, respectively, such as Gradient Boosting Decision Tree (GBDT), eXtreme Gradient Boosting (XGBoost), Stacking, Logistic Regression (LR), support vector machine (SVM), and random forest (RF), to determine the optimal predictive model for CWP.

### 2.4 Statistical analysis

The data analyses were statistically performed using IBM SPSS Statistics 26.0. Measurement data that meet the normal distribution, tested by a Student's *t*‐test, were expressed as mean ± standard deviation (x ± s); non‐normal distribution data, tested by a Wilcoxon signed‐rank test, was presented as median (M) or interquartile range (IQR). Predictive performances between different models were evaluated using the receiver operating characteristic (ROC) curve and area under the ROC curve (AUC), whose value ranges from 0 to 1; larger AUC values represent better performances than the algorithm predicted. Statistical significance was defined at a value of *p* below 0.05.

## 3 RESULTS

### 3.1 Patients' characteristics

During 28 August 2021 until 12 December 2021, 52 patients with CWP (Stage I) and 58 dust‐exposed workers, belonging to male patients aged 33–70 years with cough, dyspnoea, or other symptoms, were enrolled in this study in the dust‐exposed workers' group (the average age was 49.8 ± 7.3 years old), whose exposure duration was 25.6 ± 7.2 years, while in the CWP (Stage I) group (the average age was 59.8 ± 6.4 years old), whose exposure duration was 28.8 ± 5.7 years.

### 3.2 Model performances of different feature selection methods

#### 3.2.1 Lasso CV regression analysis prediction

**FIGURE 2:**
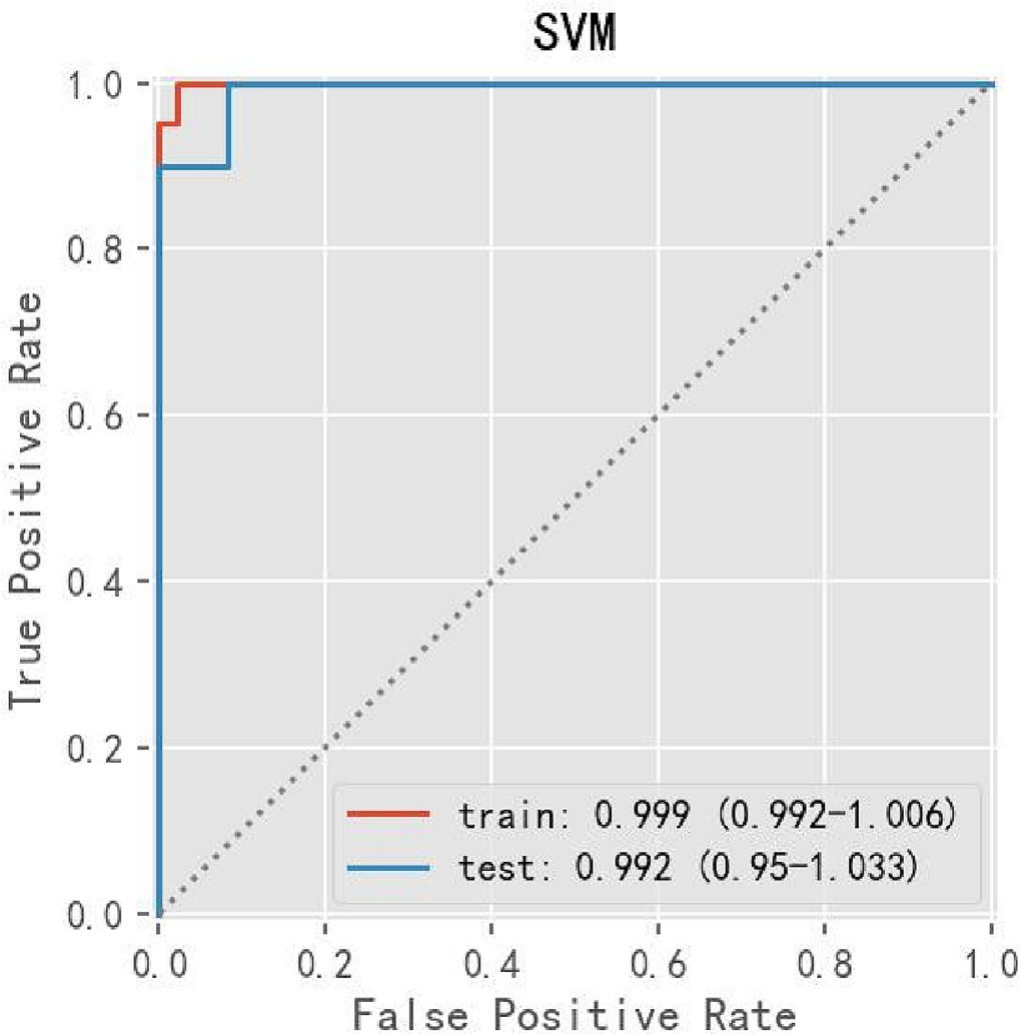
The performance of SVM evaluated by ROC curve in Lasso CV regression analysis. SVM, support vector machine.

To build a robust predictive model, Lasso CV regression was employed for its stability to construct the prediction model, performing feature selection. On the basis of the previous feature selection, we obtained 20 clinical parameters and thus performed different machine learning models (RF, LR, SVM, GBDT, XGBoost, Stacking) training to determine the optimal predictive model. The comparison results of different ML algorithms indicated that applying the SVM algorithm accomplished this goal of obtaining the optimal model parameters (gamma = 0.1) by using a grid search. We observed the best performance for the SVM model, significantly better than other models; for this condition, the accuracy was 93.9%, sensitivity was 100%, specificity was 89%, and AUC was 0.992, as demonstrated in Table 2 and Figure 2.

**TABLE 2:** The comparison results of different algorithms in the LASSO regression model.

	Train_Accuracy	Test_Accuracy	Specificity	Sensitivity	AUC	F1–Score
LR	1.000	0.848	0.857	0.8	0.955	0.827
RF	1.000	0.909	0.928	0.867	0.981	0.896
SVM	1.000	0.939	0.882	1.000	0.992	0.937
GBDT	1.000	0.878	0.923	0.8	0.959	0.857
XGBoost	1.000	0.939	0.933	0.933	0.978	0.933
Stacking	1.000	0.909	0.928	0.867	0.981	0.896

Abbreviations: GBDT, Gradient Boosting Decision Tree; LR, Logistic Regression; RF, random forest; SVM, support vector machine; XGBoost, eXtreme Gradient Boosting.

#### 3.2.2 Univariate analysis prediction

**FIGURE 3:**
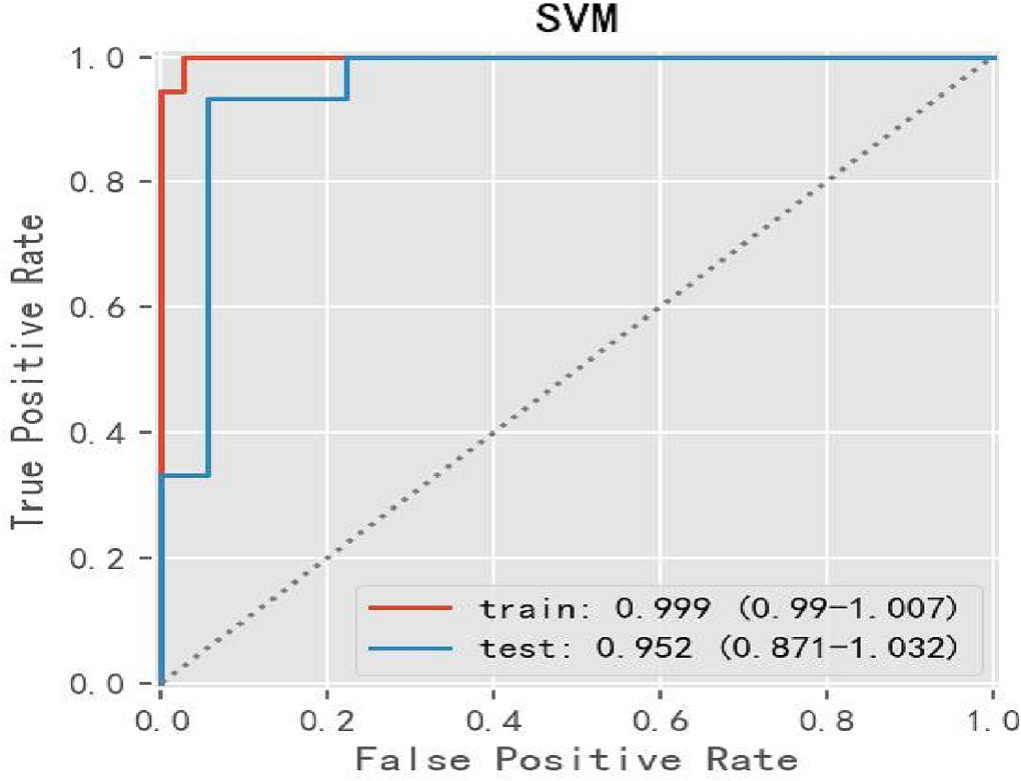
The performance of SVM evaluated by ROC curve in univariate analysis. SVM, support vector machine.

After univariate analysis of the 62 clinical parameters (*p* < 0.05), 16 clinical parameters were selected that showed statistically significant predictors. We constructed different predictive models by using different machine learning models (RF, LR, SVM, GBDT, XGBoost, Stacking) by these 16 parameters; after multiple comparisons, SVM still achieved the best result among these ML‐models, for this condition, the accuracy was 93.9%, sensitivity was 93.3%, specificity was 94%, and AUC was 0.952, as demonstrated in Table 3 and Figure 3.

**TABLE 3:** The comparison results of different algorithms in the univariate analysis model.

	Train_Accuracy	Test_Accuracy	Specificity	Sensitivity	AUC	F1–Score
LR	0.948	0.909	0.875	0.933	0.959	0.903
RF	0.948	0.909	1.000	0.800	0.989	0.889
SVM	0.974	0.939	0.933	0.933	0.952	0.933
GBDT	1.000	0.879	0.923	0.800	0.956	0.857
XGBoost	1.000	0.939	1.000	0.867	0.996	0.929
Stacking	1.000	0.939	1.000	0.867	0.956	0.929

Abbreviations: GBDT, Gradient Boosting Decision Tree; LR, Logistic Regression; RF, random forest; SVM, support vector machine; XGBoost, eXtreme Gradient Boosting.

#### 3.2.3 Boruta feature selection analysis prediction

In our pre‐experiments, the result indicated that the prediction performance of Boruta feature selection analysis prediction, with respect to the comparison among machine learning models, is unstable, with the accuracy ranging from 0.72 to 0.85, much lower than that in the two previous models, which implied that the construction of this model seemed volatile. According to existing literature,^24^ Boruta, generally based on tree model analysis, has been applied in medical fields. So we identified 13 essential features through the Boruta feature selection method using random forest analysis significantly; this combined model was applied to evaluate the predicted significance of these clinical indicators for clinical diagnosis of CWP and compared the results for three feature selection methods through the feature importance of random forest.

#### 3.2.4 Results of random forest of the feature importance evaluation with three feature selection methods

**FIGURE 4:**
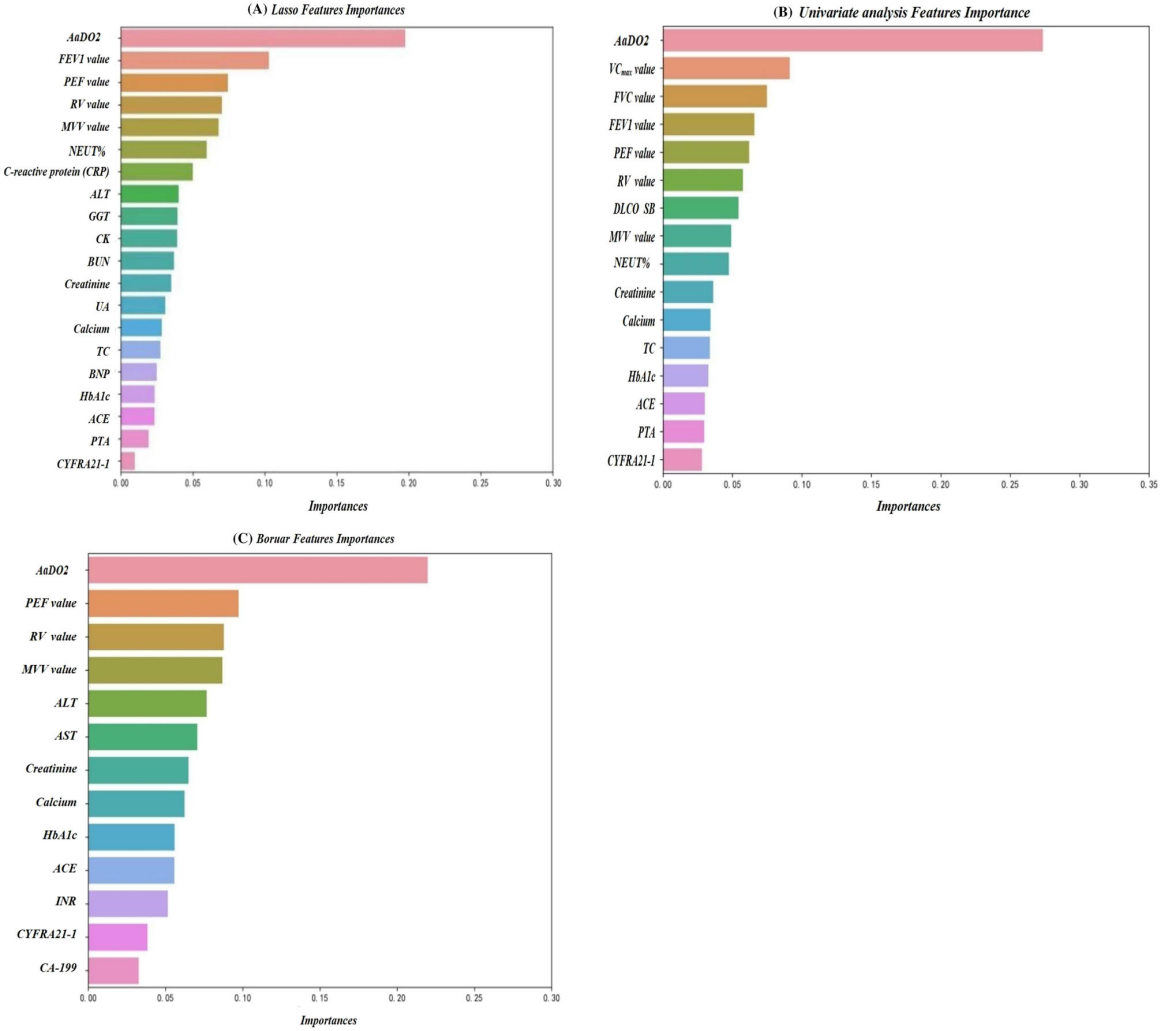
Feature importance evaluation with different feature selection methods. Feature importance evaluation with (A) Lasso feature selection method. (B) Univariate selection method. (C) Boruta selection method.

Random forest could be used to verify the importance of characteristics of clinical data. To obtain good predicted factors for CWP, we compared the results for three feature selection methods through the feature importance of random forest, as described in Figure 4. Among these three models, AaDO_2_ was all demonstrated to be vital. Besides, it was implied that the top five feature importance with high normalized predicted factors, including PEF‐value, RV, FEV1‐value, and MVV‐value, from the comparison of three models, might be predictive factors with greater relevance to CWP, which was in agreement with previous findings demonstrating that the pneumoconiosis severity might be positively correlated with the pulmonary function level.^25^, ^26^

#### 3.2.5 SVM algorithm's evaluation through ROC curves of random forest

**FIGURE 5:**
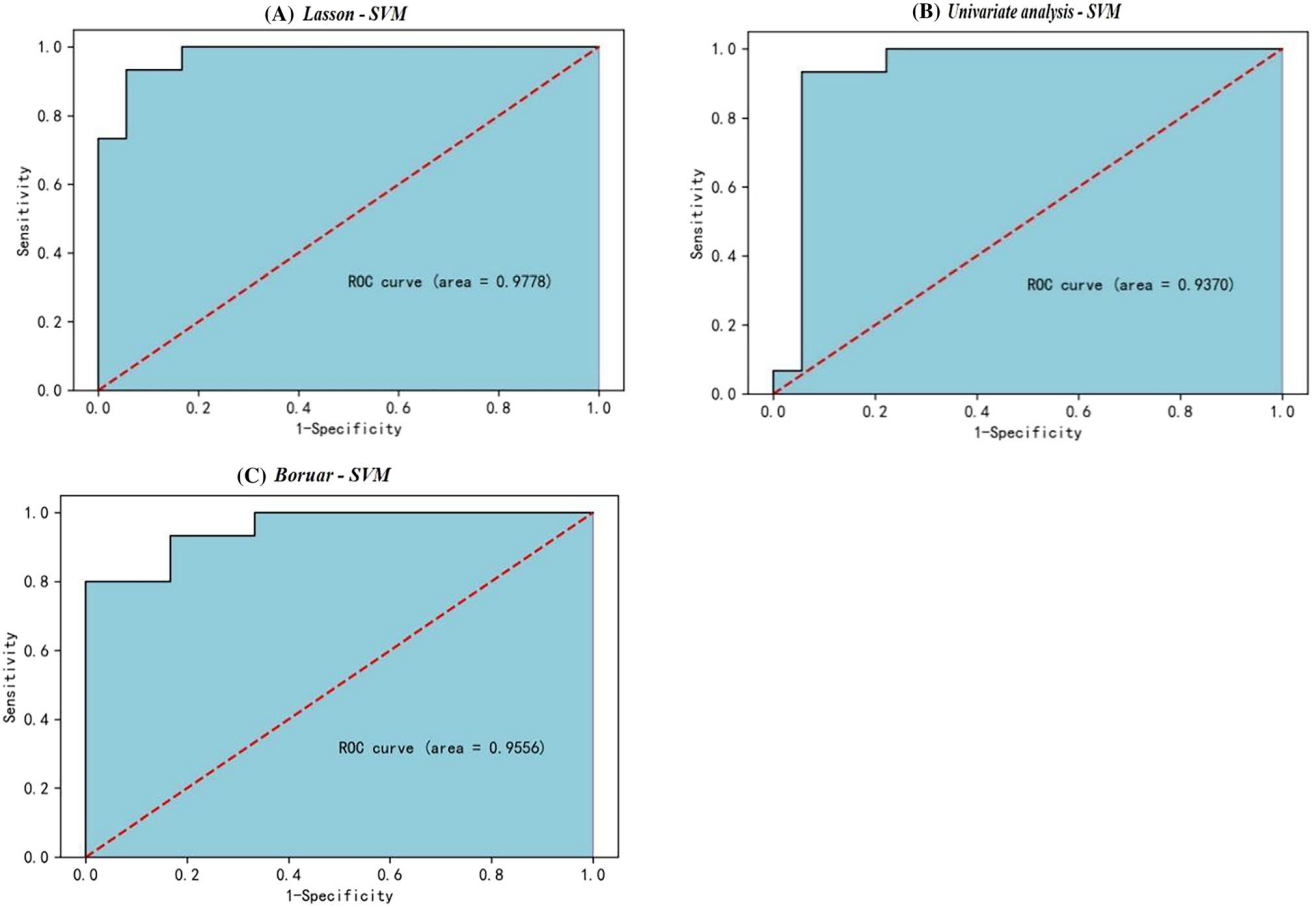
SVM algorithm's evaluation through ROC curves. (A) ROC curves of Lasso‐SVM. (B) ROC curves of univariate‐SVM. (C) ROC curves of Boruta‐SVM.

The ROC curves obtained from three feature selection methods (Lasso analysis, univariate analysis, and Boruta analysis) using the SVM algorithm are shown in Figure 5, with AUROC of 97.78%, 93.7%, and 95.56%, respectively, and the SVM was determined as the best machine learning method for predicting CWP in this study.

## 4 DISCUSSION

In our research, concerning the three feature selection approaches (Lasso CV regression, Boruta feature selection, and univariate analysis), which were operated with different supervised machine learning algorithms, through comparing the performances of the different machine learning algorithms used herein, we obtained the optimal feature selection/machine learning algorithm as a novel and reliable tool for predicting the risk of CWP in a clinical environment. After close data analysis and careful evaluation of machine learning algorithms, clinical indicators (arterial blood gas analysis, pulmonary function test, blood cell analysis, inflammatory markers, blood biochemical parameters, coagulation function, and serum tumor markers) belonged to common and significant predictors of pulmonary disease diagnosis. Our results revealed that AaDO_2_ (arterial blood gas analysis) and some indicators of pulmonary function test, including PEF‐value, RV, FEV1‐value, and MVV, ranked in the top five feature importance predicted factors, were significant predictors of CWP early diagnosis clinically; however, these remains to need additional analyses to confirm our conclusion in the future.

Pulmonary function and blood gas analysis were represented as good indicators of the capability of pulmonary ventilation and pulmonary gas exchange. In the occupational health field, both of the methods mentioned above could reflect the severity of pulmonary damage caused by pneumoconiosis.^25^, ^26^ Pulmonary function testing belonged to a simple, safe, and inexpensive modality and was indicated for assessing the status and severity of lung disease and screening of pulmonary disorders. Compared to X‐ray and CT, it may directly reflect lung function, including pulmonary gas exchange and pulmonary ventilation function.^27^ Previous studies have disclosed that pulmonary function can be helpful in the early evaluation of patients with pneumoconiosis. Blood gas analysis can be used to evaluate the acid–base status, oxygenation, and ventilation clinically,^28^, ^29^ which was a feasible method to reflect lung respiratory function directly; however, its application in the diagnosis of early‐stage pneumoconiosis was still a matter of debate. Benefiting from the powerful compensatory function of the lungs, hypoxemia (PaO2 < 60 mmHg) measured by the blood gas analysis may generally be present in the middle and the advanced stages of pneumoconiosis. This might result in pulmonary function appeared already in the abnormal range at the time of the disease assessment, while blood gas analysis was still in a normal state, which indicated substantial variability in the results between different clinical analyses.

From our study findings, AaDO_2_ is a more sensitive indicator for assessing various clinical indicators' predicting ability in CWP. Pulmonary fibrosis, resulting from pneumoconiosis, could induce airway narrowing and lead to alveolar hypoventilation, thus resulting in a reduced area of lung gas diffusion, marked structural abnormalities of the alveolar–capillary interface, long‐term impairment of gas exchange, and a higher alveolar‐arterial oxygen pressure difference (AaDO_2_). There was an abnormal increase in the value of AaDO_2_ with higher stages of pneumoconiosis, which was concordant with the findings of this study.^30^ The design idea of our research was from a clinical point of view, mainly focusing on analyzing clinical indicators data using machine learning algorithms to probe indicators with better sensitivity and specificity in predicting disease progression in CWP patients of the early phase. Several reasons for our results are presented as follows: (1) To the best of our knowledge, there are no previous studies associating analysis relevant clinical indicators for clinical diagnosis of pneumoconiosis; however, patients with CWP and dust‐exposed workers may experience nonspecific symptoms such as cough, chest tightness, and dyspnoea on exertion. Hence, analyzing relevant clinical indicators is usually the optimal option to provide comprehensive assessments for further evaluation, diagnosis, and treatment. (2) In addition, it has been suggested that CWP of the early stage can occur at any stage during the process of dust‐exposed workers.^7^ For this reason, the prediction and early diagnosis of CWP clinically are very vital. However, a reliable method or model to analyze clinical data comprehensively for the prediction of CWP is lacking.

In the study, we made use of different feature selection analysis predictions to assess the conventional clinical indicators, obtaining the prediction relevance of CWP among different clinical indicators; then compared the results of different ML algorithms in these feature selection methods, constructing an effective and relatively reliable model, acquiring the optimal machine learning algorithm (SVM) combining feature selection approaches for prediction.

We recognize several study limitations. First, our sample size may have been too small to perform a more detailed correlation analysis for machine learning. Although CWP, as an occupational disease, is relatively uncommon, and this study had adequate power for correctly interpreting the results, more analyses with increased sample sizes are required to confirm current findings. Secondly, although the SVM model could provide significantly better accuracy for identifying between CWP of early‐stage and undiagnosed dust‐exposed workers, the present study did not address the relationship among different stages of CWP, which deserved further investigation. Thirdly, there was no information or inaccurate information on smoking, body mass index (BMI), or other potential influencing factors, presenting challenges to the comprehensive analysis of CWP. Therefore, a dataset with complete epidemiologic information would likely contribute to better model predictive performance and more reliable analysis results.

## 5 CONCLUSION

The present study applied three feature selection approaches (Lasso CV regression, Boruta feature selection, and univariate analysis) based on machine learning algorithms; we concluded that AaDO_2_ and some indicators of pulmonary function, such as PEF‐value, RV, FEV1‐value, and MVV had been found to play an essential role in prediction for identifying between CWP of early stage and undiagnosed dust‐exposed workers. Furthermore, we developed the optimal model (SVM algorithm) through comparisons and analyses among the performances of different models; thus SVM algorithm could effectively analyze clinical data comprehensively for the prediction of CWP as an actual clinical application, thus giving advantages to obtaining an early diagnosis of CWP in the clinical practice.

## AUTHORS CONTRIBUTIONS

Hantian Dong designed and conceived this research, participated in image data collection and interpretation of all data, and wrote this manuscript. Biaokai Zhu took full responsibility for the ML algorithm and statistical analysis. Xiaomei Kong was responsible for the supervision of the clinical data collection and data management and was involved in reviewing this manuscript. Xinri Zhang supervised the data quality control and data analyses, interpreted ML algorithm analyses, and critically reviewed the manuscript. All authors have read and consented to the final manuscript.

## CONFLICT OF INTEREST STATEMENT

All authors have no relevant competing interests to disclose.

## ETHICS APPROVAL AND CONSENT TO PARTICIPATE

All the patients involved in the study provided written informed consent forms. Ethical approval for the study (reference no. 2020 K‐K104) was given by the Research Ethics Committee, the First Hospital of Shanxi Medical University. Moreover, all methods used in this manuscript were implemented in accordance with relevant guidelines and regulations by the Declaration of Helsinki.

## Supporting information


**Table S1.** All clinical data of patients in the group with CWP and with dust‐exposed workers.

## Data Availability

The clinical datasets used and analyzed during the current study are not publicly available for reasons of the policies of the fund‐funded institutions; all research data of the study may be uniformly disclosed after completion but are available from the corresponding author upon reasonable request. The ML algorithm data in the article are available at GitHub online (direct link at https://github.com/HantianDong1988/Pneumoconiosis-Machine-learning-clinical-data-analysis-research).
