# Supplementary material for: Efficient clinical data analysis for prediction of coal workers' pneumoconiosis using machine learning algorithms
Source: Clin Respir J. 2023 Jun 28;17(7):684–93. doi: 10.1111/crj.13657 (PMC10363790; doi:10.1111/crj.13657)
Supplement: Supplementary file 1 — Table S1. All clinical data of patients in the group with CWP and with dust‐exposed workers. [file CRJ-17-684-s001.docx]

**Supplementary**

**Table.1** All clinical data of patients in the group with CWP and with dust-exposed workers

|  | Dust-exposed workers/Control group (n=58) | CWP Stage I (n=52) | Statistic | P-value |
| --- | --- | --- | --- | --- |
| **Arterial Blood Gas Analysis** |  |  |  |  |
| pH, mean (SD) | 7.378（0.021） | 7.381（0.019） | t =0.772 | 0.442 |
| PaCO_2_(mmHg),mean(SD) | 41.2（2.694） | 40.977（2.918） | t =0.005 | 0.996 |
| PaO_2_(mmHg),IQR | 89.9（9.07） | 85.95（7.47） | Z=0.714 | 0.475 |
| SaO_2_(%),IQR | 96.3（1.15） | 95.85（1.32） | Z=3.414 | **<0.05** |
| HCO_3_-(mmol/L),mean(SD) | 23.75（1.426） | 23.66（1.383） | t =0.075 | 0.940 |
| Oxygenation index, IQR | 428.0（43.92） | 408.5（35.36） | Z=0.407 | 0.684 |
| AaDO_2_(mmHg),IQR | 8.9（7.09） | 13.51（6.43） | Z=2.128 | **<0.05** |
|  |  |  |  |  |
| **Pulmonary function test** |  |  |  |  |
| VCmax-value(L),IQR | 4.19（1.02） | 3.72（0.63） | Z=0.383 | 0.702 |
| FVC-value(L),IQR | 4.09（1.06） | 3.635（0.55） | Z=0.344 | 0.731 |
| FEV1-value(L),IQR | 3.14（0.94） | 2.765（0.53） | Z=0.467 | 0.641 |
| FEV1/FVC(%),IQR | 79.34（6.41） | 77.595（8.17） | Z=0.163 | 0.871 |
| PEF-value(L/s),mean(SD) | 8.57（1.349） | 7.53（1.255） | t =0.004 | 0.997 |
| RV-value(L),IQR | 2.05（0.48） | 2.25（0.33） | Z=0.13 | 0.897 |
| TLC-value(L),IQR | 5.97（1.21） | 5.645（0.89） | Z=0.443 | 0.658 |
| DLCO-SB (mmol/min/Kpa), mean(SD) | 9.04（1.337） | 8.207（1.314） | t =0.067 | 0.946 |
| DLCO/VA  -SB(mmol/min/Kpa/L),mean(SD) | 1.55（0.195） | 1.484（0.252） | t =0.089 | 0.93 |
| MVV-value(L/min),IQR | 107.4（23.43） | 91.33（24.57） | Z=0.926 | 0.354 |
| IC, IQR | 3.01（0.53） | 2.89（0.66） | Z=0.55 | 0.582 |
| FeNO-value(ppb),IQR | 22.0（8.5） | 25.5（12.5） | Z=1.615 | 0.106 |
| **Blood cell analysis** |  |  |  |  |
| WBC Count(×10^9^/L),IQR | 5.5（2.02） | 6.05（2.0） | Z=0.744 | 0.457 |
| NEUT%, mean(SD) | 58.98（7.775） | 63.431（7.814） | t =0.003 | 0.998 |
| Total count of blood lymphocytes(×10^12^/L),IQR | 1.6（0.5） | 1.6（0.7） | Z=1.243 | 0.214 |
| Total count of blood monocytes(×10^12^/L),IQR | 0.4（0.2） | 0.4（0.2） | Z=1.14 | 0.254 |
| Eosinophil Count(×10^12^/L),IQR | 0.2（0.27） | 0.1（0.42） | Z=1.395 | 0.163 |
| Hemoglobin Level(g/L),mean(SD) | 159.89（9.92） | 162.23（11.65） | t =0.007 | 0.994 |
| Platelet(×10^9^/L),IQR | 223.5（101.5） | 213（45.75） | Z=0.401 | 0.688 |
| Basophil Count(×10^12^/L),IQR | 0.1（0.4） | 0.2（0.4） | Z=0.983 | 0.326 |
| **Inflammatory markers** |  |  |  |  |
| ESR(mm/1_st_ h),IQR | 4.0（4.75） | 6.5（4.0） | Z=2.349 | **<0.05** |
| CRP(mg/L),IQR | 2.59（2.02） | 3.12（1.69） | Z=1.725 | **<0.05** |
| **Blood biochemical parameters** |  |  |  |  |
| ALT(IU/L),IQR | 29.0（14.75） | 21（10.25） | Z=2.635 | **<0.05** |
| AST(IU/L),IQR | 27.0（8.5） | 22（5.25） | Z=3.242 | **<0.05** |
| Total bilirubin (TBil)(umol/L),IQR | 14.85（6.85） | 15.05（7.67） | Z=1.391 | 0.164 |
| GGT(IU/L),IQR | 31（20.25） | 38.5（73.25） | Z=2.951 | **<0.05** |
| ALP(IU/L),IQR | 91.0（31.5） | 93.5（25.5） | Z=0.75 | 0.453 |
| CK(IU/L),IQR | 110.0（61.75） | 96.5（30.5） | Z=1.848 | **<0.05** |
| BUN(mmol/L),IQR | 5.175（1.79） | 5.905（1.76） | Z=0.264 | 0.792 |
| Creatinine(umol/L),mean(SD) | 66.69（8.52） | 74.05（9.47） | t =0.077 | 0.939 |
| UA(umol/L),IQR | 339.5（117） | 345（90.0） | Z=0.497 | 0.619 |
| K^+^(mmol/L),IQR | 3.7（0.3） | 3.7（0.35） | Z=0.489 | 0.625 |
| Na^+^(mmol/L),IQR | 137（2.0） | 137（3.0） | Z=0.498 | 0.619 |
| Cl^－^(mmol/L),IQR | 112（3.0） | 112（2.0） | Z=0.447 | 0.655 |
| Ca^2+^(mmol/L),IQR | 1.125（0.03） | 1.1（0.12） | Z=2.335 | **<0.05** |
| TC(mmol/L),IQR | 4.62（1.2） | 5.325（1.46） | Z=0.559 | 0.576 |
| TG(mmol/L),IQR | 1.5（1.4） | 2.065（2.04） | Z=1.421 | 0.155 |
| HDL(mmol/L),IQR | 1.14（0.37） | 1.12（0.3） | Z=0.869 | 0.385 |
| LDL(mmol/L),IQR | 2.93（1.06） | 3.275（1.1） | Z=0.33 | 0.742 |
| BNP(pg/ml),IQR | 9.0（10.5） | 12（12.0） | Z=1.959 | **<0.05** |
| HbA1c(%),IQR | 4.63（0.46） | 5.44（0.7） | Z=2.367 | **<0.05** |
| ACE(IU/ml),IQR | 27.9（17.65） | 41.6（40.0） | Z=1.233 | 0.218 |
| **Coagulation function** |  |  |  |  |
| PT(s),IQR | 11.6（0.7） | 11.9（0.5） | Z=0.795 | 0.426 |
| PTA(%),IQR | 93.8（13.6） | 88.5（8.6） | Z=0.7 | 0.484 |
| INR, IQR | 1.01（0.07） | 1.04（0.04） | Z=2.777 | **<0.05** |
| APTT(s),mean(SD) | 26.812（2.693） | 27.32（3.607） | t =0.175 | 0.861 |
| Fib(mg/dl),IQR | 2.55（0.68） | 2.9（0.89） | Z=0.804 | 0.422 |
| D-dimer(mg/L),IQR | 0.18（0.19） | 0.26（0.25） | Z=2.623 | **<0.05** |
| **Serum tumor markers** |  |  |  |  |
| CEA(ng/ml),IQR | 2.12（2.31） | 2.125（2.05） | Z=1.508 | 0.132 |
| NSE(ng/ml),mean(SD) | 8.633（2.536） | 8.048（2.861） | t =0.22 | 0.826 |
| SCC(ug/L),IQR | 0.71（0.26） | 0.73（0.42） | Z=1.359 | 0.174 |
| CYFRA21-1(ng/ml),IQR | 1.32（0.52） | 1.8（1.01） | Z=1.323 | 0.186 |
| CA19-9(U/ml),IQR | 5.27（3.79） | 9.195（9.65） | Z=3.258 | **<0.05** |
| CA125(U/ml),IQR | 11.33（3.23） | 10.925（3.33） | Z=1.227 | 0.220 |

Bold characters represent statistical significance. Values are given as median (lower quartile, upper quartile) or n (percent). Abbreviations:PH, Hydrogen ion concentration; PaCO2, Arterial carbon dioxide tension; PaO2, Arterial partial pressure of oxygen; SaO2, Arterial blood oxygen saturation; HCO3-, Bicarbonate; AaDO2, Alveolar-arterial oxygen difference; VCmax-value, Max value of vitalcapacity; FVC-value, Value of forced vital capacity; FEV1-value, Value of the forced expiratory volume in the first second; RV-value, Value of residual volume; TLC-value, Value of the total lung capacity; DLCO-SB, Single breath of lung diffusing capacity for carbon monoxide; DLCO/VA-SB, Single breath of diffusion capacity for carbon monoxide per liter of alveolar volume; MVV-value, Value of maximal voluntary ventilation; IC, Inspiratory capacity; FeNO-value, Value of Fraction of Exhaled Nitric Oxide; WBC, white blood cell; NEUT%, percentage of neutrophils; ESR, Erythrocyte sedimentation rate; CRP, C-reactive protein; ALT, Alanine transaminase; AST, Aspartate aminotransferase; GGT, Gamma glutamyl transpeptidase; ALP, Alkaline phosphatase; CK, Creatine kinase; BUN, Blood urea nitrogen; TC, Serum total cholesterol; TG, Triglyceride; HDL, High density lipoprotein; LDL, Low density lipoprotein; BNP, B-natriuretic peptide; HbA1c, Hemoglobin A1c; ACE, Angiotensin I-converting enzyme; PT, prothrombin time; PTA, Prothrombin time activity; INR, international normalized ratio; APTT, Activated partial thromboplastin time; Fib, fibrinogen; CEA, Carcinoembryonic antigen; NSE, Neuron-specific enolase; SCC, Squamous cell carcinoma antigen; CYFRA21-1, Cytokeratin 19 fragment antigen 21-1; CA19-9, Carbohydrate Antigen 19-9; CA125, Carbohydrate Antigen 125.
